# Standardization of brain MR images across machines and protocols: bridging the gap for MRI-based radiomics

**DOI:** 10.1038/s41598-020-69298-z

**Published:** 2020-07-23

**Authors:** Alexandre Carré, Guillaume Klausner, Myriam Edjlali, Marvin Lerousseau, Jade Briend-Diop, Roger Sun, Samy Ammari, Sylvain Reuzé, Emilie Alvarez Andres, Théo Estienne, Stéphane Niyoteka, Enzo Battistella, Maria Vakalopoulou, Frédéric Dhermain, Nikos Paragios, Eric Deutsch, Catherine Oppenheim, Johan Pallud, Charlotte Robert

**Affiliations:** 1 Molecular Radiotherapy and Innovative Therapeutics, INSERM UMR1030, Gustave Roussy Cancer Campus, Paris-Saclay University, Villejuif, France; 2 Department of Radiotherapy, Gustave Roussy, Paris-Saclay University, 94805 Villejuif, France; 3 Department of Neuroradiology, Sainte-Anne Hospital, 75014 Paris, France; 4 Paris Descartes University, Sorbonne Paris Cité, Paris, France; 5 UMR 1266 INSERM, IMA-BRAIN, Institute of Psychiatry and Neurosciences of Paris, Paris, France; 6 Mathematics and Informatics for Complex, CentraleSupélec, Paris-Saclay University, 91190 Gif-sur-Yvette, France; 7 Department of Radiology, Paris-Saclay University, Gustave Roussy, 94805 Villejuif, France; 8 TheraPanacea, Paris, France; 9 Department of Neurosurgery, Sainte-Anne Hospital, 75014 Paris, France

**Keywords:** Diagnostic markers, Computational science, Cancer imaging, CNS cancer

## Abstract

Radiomics relies on the extraction of a wide variety of quantitative image-based features to provide decision support. Magnetic resonance imaging (MRI) contributes to the personalization of patient care but suffers from being highly dependent on acquisition and reconstruction parameters. Today, there are no guidelines regarding the optimal pre-processing of MR images in the context of radiomics, which is crucial for the generalization of published image-based signatures. This study aims to assess the impact of three different intensity normalization methods (Nyul, WhiteStripe, Z-Score) typically used in MRI together with two methods for intensity discretization (fixed bin size and fixed bin number). The impact of these methods was evaluated on first- and second-order radiomics features extracted from brain MRI, establishing a unified methodology for future radiomics studies. Two independent MRI datasets were used. The first one (DATASET1) included 20 institutional patients with WHO grade II and III gliomas who underwent post-contrast 3D axial T1-weighted (T1w-gd) and axial T2-weighted fluid attenuation inversion recovery (T2w-flair) sequences on two different MR devices (1.5 T and 3.0 T) with a 1-month delay. Jensen–Shannon divergence was used to compare pairs of intensity histograms before and after normalization. The stability of first-order and second-order features across the two acquisitions was analysed using the concordance correlation coefficient and the intra-class correlation coefficient. The second dataset (DATASET2) was extracted from the public TCIA database and included 108 patients with WHO grade II and III gliomas and 135 patients with WHO grade IV glioblastomas. The impact of normalization and discretization methods was evaluated based on a tumour grade classification task (balanced accuracy measurement) using five well-established machine learning algorithms. Intensity normalization highly improved the robustness of first-order features and the performances of subsequent classification models. For the T1w-gd sequence, the mean balanced accuracy for tumour grade classification was increased from 0.67 (95% CI 0.61–0.73) to 0.82 (95% CI 0.79–0.84, *P* = .006), 0.79 (95% CI 0.76–0.82, *P* = .021) and 0.82 (95% CI 0.80–0.85, *P* = *.*005), respectively, using the Nyul, WhiteStripe and Z-Score normalization methods compared to no normalization. The relative discretization makes unnecessary the use of intensity normalization for the second-order radiomics features. Even if the bin number for the discretization had a small impact on classification performances, a good compromise was obtained using the 32 bins considering both T1w-gd and T2w-flair sequences. No significant improvements in classification performances were observed using feature selection. A standardized pre-processing pipeline is proposed for the use of radiomics in MRI of brain tumours. For models based on first- and second-order features, we recommend normalizing images with the Z-Score method and adopting an absolute discretization approach. For second-order feature-based signatures, relative discretization can be used without prior normalization. In both cases, 32 bins for discretization are recommended. This study may pave the way for the multicentric development and validation of MR-based radiomics biomarkers.

## Introduction

Radiomics relies on the extraction of a wide variety of quantitative image-based features, including shape, histogram-based, textural and higher order statistics^1^. Along with machine learning techniques, radiomics is becoming an increasingly popular computer-aided diagnostic tool in the field of medical research^2,3^. Radiomics offers an almost unlimited supply of imaging biomarkers that can facilitate cancer detection, diagnosis, and prognosis assessment and the prediction of treatment response^1–4^.

Magnetic resonance imaging (MRI) exhibits high soft tissue contrast and submillimetre spatial resolution. In the context of radiomics, a main issue is that MRI intensities are non-standardized and are highly dependent on the manufacturer, sequence type and acquisition parameters^5^. Consequently, a large variability in image intensities among inter-patient and intra-patient acquisitions exists that could highly affect the extraction of the radiomics features, compromising the pooling and the reproducibility of published data using independent imaging sets^6,7^.

To solve this problem, previous radiomics studies have focused on image pre-processing techniques. For example, it has been shown that bias field correction efficiently minimizes MR intensity inhomogeneity within a tissue region^8–10^. The variability generated by different voxel sizes can also be reduced by spatial resampling^9,11,12^. Moreover, brain extraction is mandatory to remove the skull regions that generate the most important variations in intensities and to define the region in which intensities should be considered before any image intensity normalization^13,14^. However, even though these three types of pre-processing of brain MRI are widely accepted by the community, there is no consensus within radiomics studies regarding the applied image normalization method (Table 1). In this study, we focused on three normalization methods that were selected for their representativeness within current radiomics studies (Nyul, WhiteStripe and Z-Score). These techniques include relatively simple (e.g., Z-Score) to more complex (e.g., WhiteStripe) formulations.

**Table 1:** Normalization methods and grey level discretization applied in recent radiomics studies dedicated to brain tumors.

References	Multicenter	Number of patients	MRI sequences	Normalization technique	Grey-level discretization	Radiomics software	Features	Objective
Su et al.^15^	No	100	T2w-flair	–	–	Pyradiomics	18 first-order, 13 shape, 54 texture	Investigate the feasibility of predicting H3 K27M mutation status by applying an automated machine learning approach to the MR radiomics features of patients with midline gliomas
Liu et al.^16^	Yes	130	T1w, T2w-fl1air	ComBat	–	Artificial Intelligence Kit (GE)	First-order, texture	Develop and validate a model that can be used to predict the individualized treatment response in children with cerebral palsy
Bologna et al.^17^	–	Phantom	T1w, T2w	Z-Score	32 FBN	Pyradiomics	18 first-order, 14 shape, 75 texture	Analysis of virtual phantom for preprocessing evaluation and detection of a robust feature set for MRI-radiomics of the brain
Elsheikh et al.^18^	Yes	135	T1w, T1w-gd, T2w, T2w-flair	–	–	–	First-order, texture	Analysis of multi-stage association of glioblastoma gene expressions with texture and spatial patterns
Tixier et al.^19^	Yes	90	T1w-gd, T2w-flair	–	128 FBN	CERR	72 features (first-order, texture, shape)	Study the impact of tumor segmentation variability on the robustness of MRI radiomics features
Ortiz-Ramón et al.^20^	No	200	T1w, T2w, T2w-flair	–	32 FBN	MATLAB	114 textures	Identify the presence of ischaemic stroke lesions by means of texture analysis on brain MRI
Vamvakas et al.^21^	No	40	T1w, T1w-gd, T2w, T2w-flair	–	–	MATLAB	11 first-order, 16 texture	Investigate the value of advanced multiparametric MRI biomarker analysis based on radiomics features and machine learning classification for glioma grading
Tixier et al.^22^	Yes	159	T1w, T1w-gd, T2w-flair	–	128 FBN	CERR	286 features (first-order, shape, texture)	Evaluate the capacity of radiomics features to add complementary information to MGMT status, to improve the ability to predict prognosis
Wu et al.^23^	Yes	126	T1w, T1w-gd, T2w, T2w-flair	–	–	–	704 features (first-order, shape, texture)	Identify the optimal radiomics-based machine learning method for isocitrate dehydrogenase genotype prediction in diffuse gliomas
Artzi et al.^24^	No	439	T1w-gd	WhiteStripe	–	MATLAB	757 features (first-order, shape, texture)	Differentiate between glioblastoma and brain metastasis subtypes using radiomics analysis
Kniep et al.^25^	No	189	T1w, T1w-gd, T2w-flair	WhiteStripe	–	Pyradiomics	18 first-order, 17 shape, 56 texture	Investigate the feasibility of tumor type prediction with MRI radiomics image features of different brain metastases in a multiclass machine learning approach for patients with unknown primary lesion at the time of diagnosis
Sanghani et al.^26^	Yes	163	T1w, T1w-gd, T2w, T2w-flair	–	–	Pyradiomics	2200 features (first-order, shape, texture)	Predict overall survival in glioblastoma multiforme patients from volumetric, shape and texture features using machine learning
Liu et al.^27^	Yes	84	T2w	Z-Score	–	MATLAB	131 features (first-order, shape, texture)	Develop a radiomics signature for prediction of progression-free survival (PFS) in lower-grade gliomas and investigate the genetic background behind the radiomics signature
Peng et al.^28^	No	66	T1w-gd, T2w-flair	–	64 FBN	MATLAB	51 features (first-order, shape, texture)	Distinguish true progression from radionecrosis after stereotactic radiation therapy for brain metastases with machine learning and radiomics
Bae et al.^29^	No	217	T1w-gd, T2w-flair	WhiteStripe	–	Pyradiomics	796 features (first-order, shape, texture)	Investigate whether radiomics features based on MRI improve survival prediction in patients with glioblastoma multiforme (GBM) when they are integrated with clinical and genetic profiles
Chen et al.^30^	Yes	220	T1w, T1w-gd, T2w, T2w-flair	Nyul	–	Pyradiomics	420 features (first-order, shape, texture)	Classify gliomas combining automatic segmentation and radiomics

The technique developed by Nyúl et al*.*^31^ and further extended by Shah et al*.*^32^ is a piecewise linear histogram matching method. In particular, in this method, a standard histogram is learned from the training set and then used to linearly map the intensities of the image of interest. Shinohara et al*.*^33^ described a statistical normalization method called WhiteStripe based on the intensity values of the normal-appearing white matter (NAWM). The Z-Score method consists of subtracting the mean intensity of the entire image or a region of interest from each voxel value and dividing it by the corresponding standard deviation^34^.

To calculate second-order features, also known as texture features, a grey-level discretization step clusters similar intensity levels into bins to minimize the noise impact and decrease calculation times^35^. This is an additional critical pre-processing step that does not express any consensus in the literature, and it is usually not truly clarified in radiomics studies (Table 1). Conventionally, the grey-level discretization can be defined as absolute if a fixed bin size (FBS) is used to cluster the intensities of the region of interest (ROI) or as relative when a fixed bin number (FBN), whose size depends on the minimum and maximum values within the same ROI, is preferred.

Even if several studies have shown variabilities in texture analysis depending on MRI acquisition parameters and the grey-level discretization step, none of them has assessed the combined impact of intensity normalization and grey-level discretization pre-processing methods on radiomics feature values in MRI^36–40^.

The objective of this study was to assess the impact of three intensity normalization methods coupled with two methods for grey-level discretization on the challenging task of tumour grade classification in two independent cohorts. Finally, we propose recommendations to standardize the pre-processing techniques of brain MRI, which is crucial to achieve reliable radiomics-based machine learning models.

## Results

### Impact of the intensity normalization method on histograms and first-order features

Jensen–Shannon divergence (JSD) values showed significant differences (*P* < 0.001) related to the intensity normalization process for both T1w-gd and T2w-flair sequences (DATASET1). On post hoc analysis, significantly higher JSD values were found when comparing images without normalization to Nyul (*P* < 0.001), WhiteStripe (*P* < 0.001) and Z-Score (*P* < 0.001) pre-processed images (Table 2). The numbers of first-order features defined as robust between the two acquisitions, depending on the normalization method, are summarized in Table 3 (DATASET1). Nyul’s method provided the highest number of robust first-order features based on a threshold value of 0.80 for both intra-class correlation coefficients (ICCs) and concordance correlation coefficients (CCCs) for both T1w-gd and T2w-flair sequences with 16 and 8 features out of 18, respectively. Images without any normalization did not generate any robust feature for the T1w-gd and T2w-flair sequences.

**Table 2:** Jensen–Shannon divergences on DATASET1 compared using a Turkey HSD test.

		Turkey HSD (mean difference)	
		T1w-gd	T2w-flair
Pair 1	No normalization-Nyul	− 0.469*	− 0.284*
Pair 2	No normalization-WhiteStripe	− 0.446*	− 0.237*
Pair 3	No normalization-Z-score	− 0.433*	− 0.241*
Pair 4	Nyul-WhiteStripe	0.024	0.048
Pair 5	Nyul-Z-score	0.036	0.043
Pair 6	WhiteStripe-Z-score	0.012	− 0.005
ANOVA	*P* value	< 0.001	< 0.001

*Significant (*P* < .05).

**Table 3:** Number of first-order features with ICCs and CCCs > 0.80 on DATASET1.

	Number of first-order features with ICCs and CCCs > 0.80	
	T1w-gd	T2w-flair
No normalization	0/18	0/18
Nyul	16/18	8/18
WhiteStripe	5/18	1/18
Z-Score	9/18	1/18

**Figure 1:**
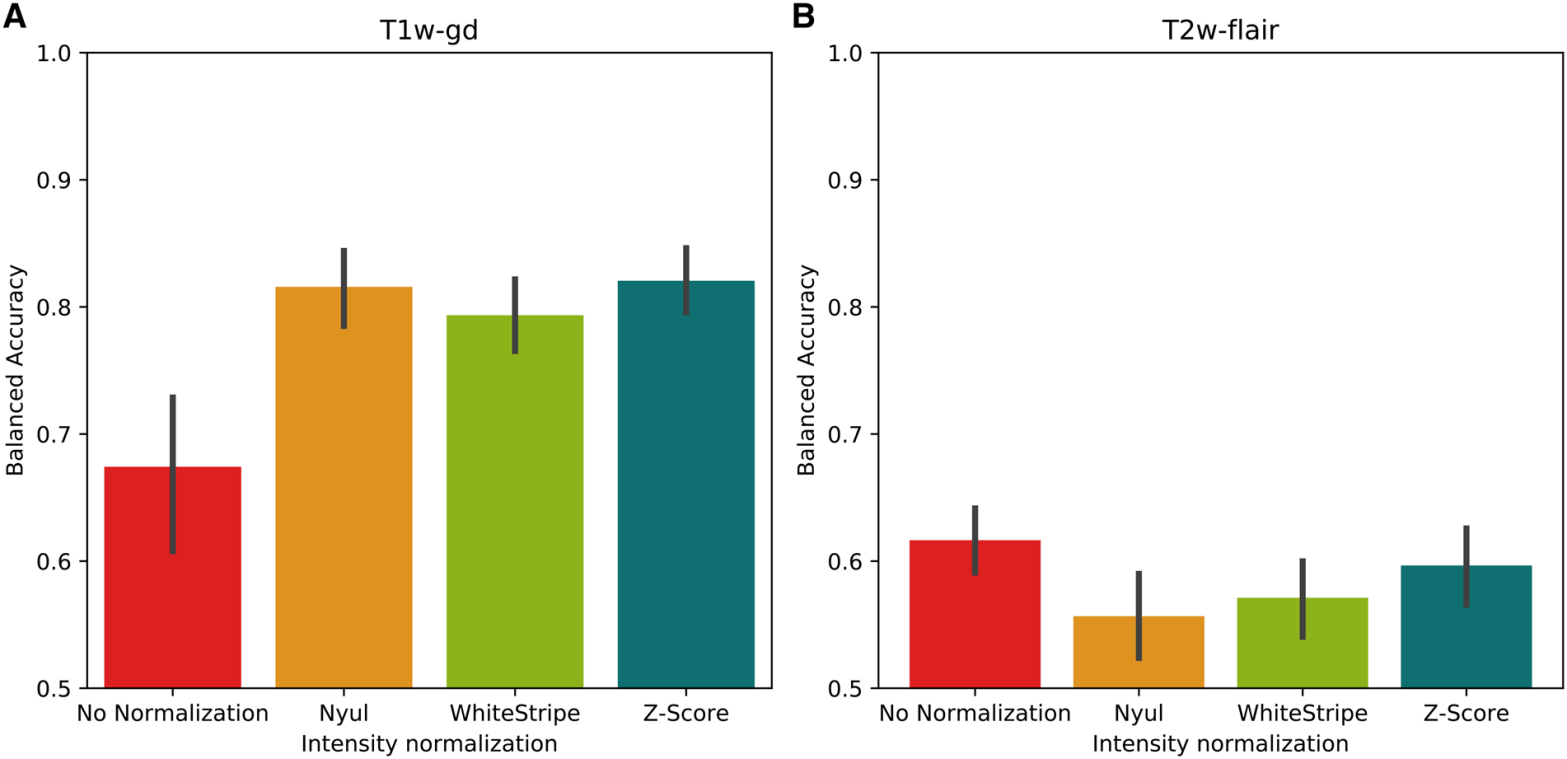
Balanced accuracies obtained for the tumour grade classification task using the 18 first-order features only. Bar plots and associated error bars represent the average balanced accuracies and the 95% CIs obtained using all 5 test folds of the cross-validation of the 5 machine learning models as a function of the normalization method, respectively. (**A**) T1w-gd MRI sequence only, (**B**) T2w-flair MRI sequence only.

For the T1w-gd sequence, the average balanced accuracy corresponding to the binary tumour grade classification task obtained from the 5 test folds and the five machine learning models using the 18 first-order features only (model 1) was equal to 0.67 (95% confidence interval (CI) 0.61–0.73) when no normalization was applied. In comparison, this value was equal to 0.82 (95% CI 0.79–0.84, *P* = 0.006), 0.79 (95% CI 0.76–0.82, *P* = 0.021) and 0.82 (95% CI 0.80–0.85, *P* = 0.005) when applying the Nyul, WhiteStripe and Z-Score pre-processing methods, respectively (DATASET2) (Fig. 1A). For the T2w-flair sequence, this value was equal to 0.62 (95% CI 0.59–0.64) when no normalization was applied and 0.56 (95% CI 0.52–0.59, *P* = 0.045), 0.57 (95% CI 0.54–0.60, *P* = 0.164), 0.60 (95% CI 0.57–0.63, *P* = 0.770) when the Nyul, WhiteStripe and Z-Score methods were applied, respectively (Fig. 1B).

### Impact of the intensity normalization method and grey-level discretization on textural features

#### Fixed bin number (FBN)

**Figure 2:**
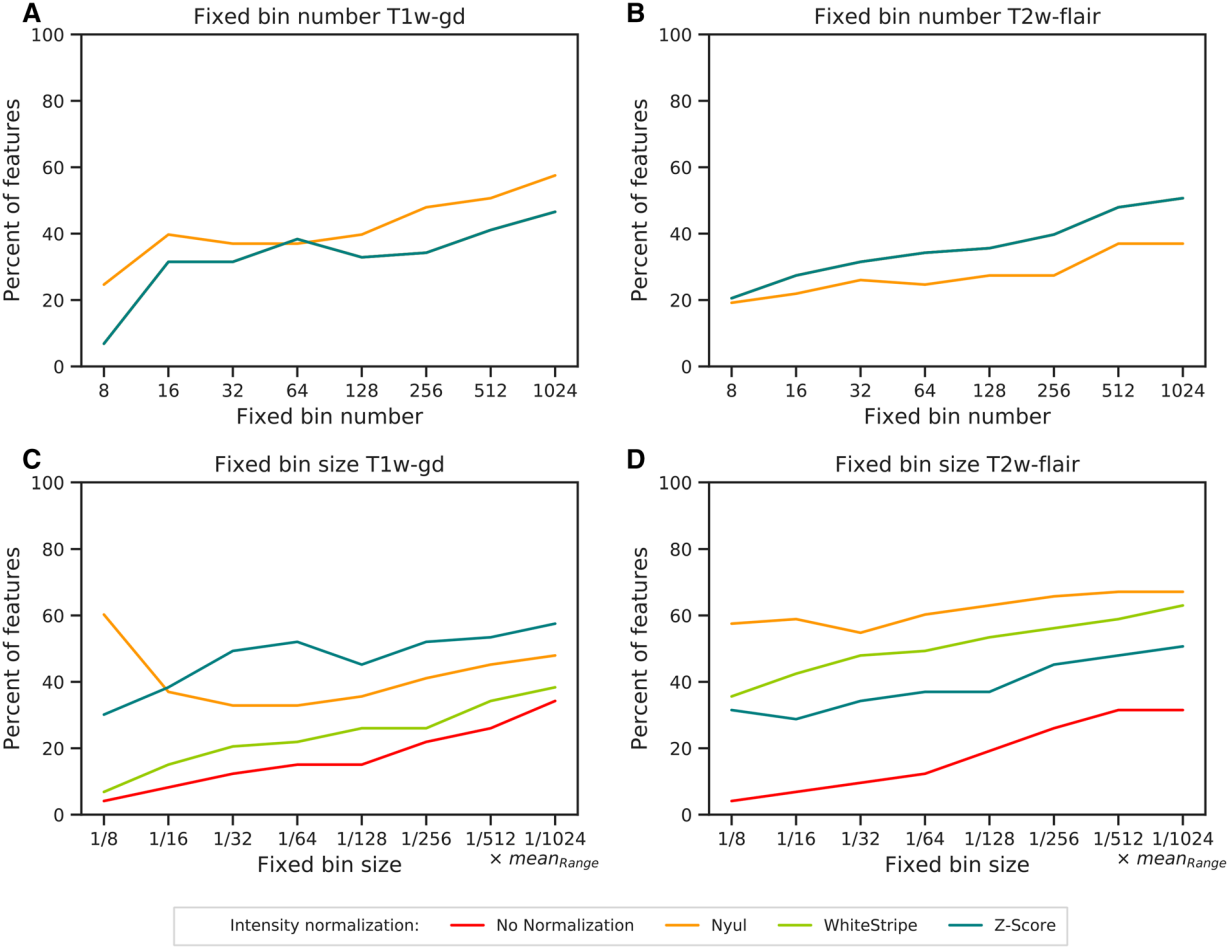
Percentages of the 73 textural features showing ICCs and CCCs values > 0.8 depending on the intensity normalization and the discretization method. (**A**) FBN T1w-gd, (**B**) FBN T2w-flair, (**C**) FBS T1w-gd, (**D**) FBS T2w-flair. *FBN* fixed bin number (relative discretization), *FBS* fixed bin size (absolute discretization), *ICC* intra-class correlation coefficient, *CCC* cross correlation coefficient. In (**A**) and (**B**), the No Normalization, WhiteStripe and Z-Score line plots are confounded. In (**C**) and (**D**), the No Normalization, WhiteStripe and Z-Score line plots are separated.

Figure 2 illustrates the percentage of the 73 textural features showing ICCs and CCCs higher than 0.8 depending on the intensity normalization and discretization method based on DATASET1. When a relative discretization was used (FBN), the WhiteStripe and Z-Score methods extracted the same feature values as the raw images, which explains the similar plots (Fig. 2A,B). Nyul’s method provided the highest percentage of robust textural features compared to images without any normalization for the T1w-gd sequence, with a mean difference of 8 percentage points (Fig. 2A) for all discretization values. For the T2w-flair sequence, features extracted from original images were more robust than those obtained by Nyul’s method (Fig. 2B). Between 16 and 128 bins, the percentages of robust features were quite stable, with a maximum variation of 10 percentage points regardless of the sequence and normalization method (Fig. 2A,B).

**Figure 3:**
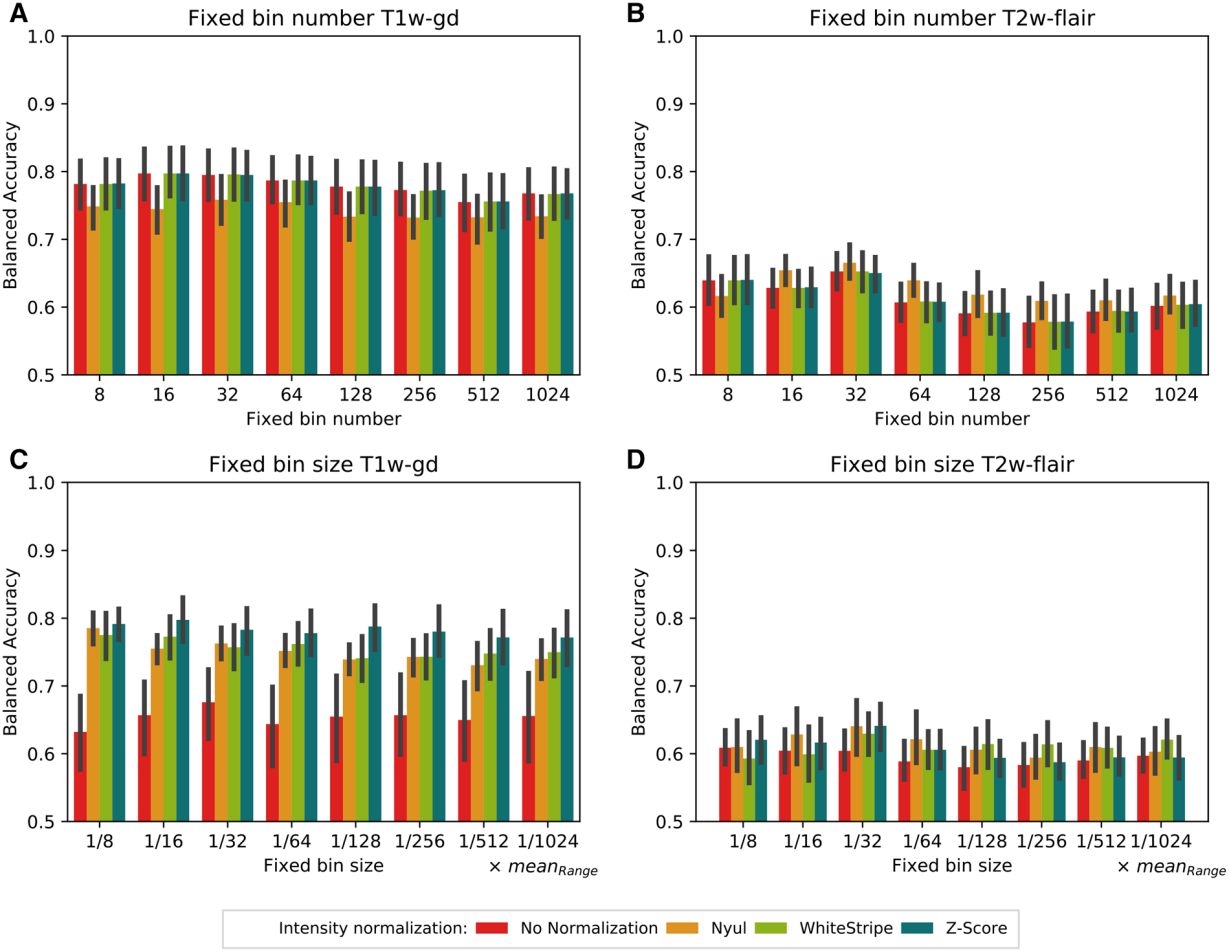
Balanced accuracies obtained for the tumour grade classification task using the 73 textural features only. Bar plots and associated error bars represent the average balanced accuracies and the 95% CIs obtained using all 5 test folds of the cross-validation of the 5 machine learning models as a function of the normalization method and number of bins, respectively. (A) FBN T1w-gd. (**B**) FBN T2w-flair. (**C**) FBS T1w-gd. (**D**) FBS T2w-flair. fixed bin number (relative discretization). *FBS* fixed bin size (absolute discretization).

Figure 3 shows the mean balanced accuracies obtained from the five machine learning models trained on the tumour grade classification task (DATASET2) using the 73 textural features only (model 2) for different intensity normalization and discretization methods. No normalization and the WhiteStripe or Z-Score methods led to the same classification performances (Fig. 3A,B). Nyul’s method resulted in 5% lower performances on average than no normalization when considering the T1w-gd sequence and all numbers of bins (Fig. 3A). Even if the ANOVA test resulted in a *P* value < 0.001 regarding the normalization effect, the difference was not statistically significant when a subsequent pairwise post hoc Tukey’s multiple comparison test was performed (*P* = 1.0). Regarding the number of bins, only the comparison between 32 and 512 bins demonstrated statistical significance (*P* = 0.039). For the T2w-flair sequence, the best classification performance was obtained using Nyul’s histogram harmonization and 32 bins, with a mean balanced accuracy of 0.67 (95% CI 0.64–0.69—Fig. 3B). No significant difference was identified regarding the impact of normalization (*P* = 0.198) as opposed to the impact of discretization (*P* < 0.001). Statistically significant results depending on the number of bins were equal to *P* = 0.012 (8–256 bins), *P* = 0.010 (32–1024 bins), *P* = 0.009 (16–256 bins), *P* = 0.001 (32–128 bins), and *P* < 0.001 (32–256 bins, 32–512 bins).

#### Fixed bin size (FBS)

When an absolute discretization was adopted, all normalization methods improved the number of robust features compared to no normalization, irrespective of the MR sequence (Fig. 2C, D—DATASET1). A higher number of bins was often associated with a higher number of robust features in both T1w-gd and T2w-flair MRI sequences. In particular, a maximum increase of 30 percentage points was reported for the T1w-gd sequence when the number of bins varied from 8 to 1024 (no normalization). For the T1w-gd sequence, considering a number of bins equal to 32, the Nyul, WhiteStripe and Z-Score methods achieved 33%, 21% and 49% robust features, respectively; regarding the T2w-flair sequence, these values were equal to 55%, 48% and 34%, respectively, for the same bin size.

The use of an MR intensity normalization method significantly improved the balanced accuracy in DATASET2 for the T1w-gd sequence (*P* < 0.001—Fig. 3). At 32 bins, the mean balanced accuracy for tumour grade classification using only textural features from the T1w-gd sequence (model 2) was equal to 0.68 (95% CI 0.62–0.72) without normalization (Fig. 3C). The same metric reached 0.76 (95% CI 0.74–0.79, *P* < 0.001), 0.76 (95% CI 0.73–0.79, *P* < 0.001), and 0.78 (95% CI 0.75–0.81, *P* < 0.001) when the Nyul, WhiteStripe and Z-Score methods were applied, respectively. Absolute improvement was poor for the T2w-flair sequence and did not exceed 0.04 for comparisons of no normalization and the Z-Score method (Fig. 3D). No significant differences were observed between the different bin numbers for T1w-gd (*P* = 0.909) and T2w-flair (*P* = 0.597) sequences.

### Performance comparison of different classification models

Table 4 summarizes the mean balanced accuracy and the area under the receiver operating characteristic curve (ROC-AUC) obtained using 32 bins for the T1w-gd and T2w-flair sequences based on first-order features only (model 1), second-order features only (model 2), first- and second-order features (model 3) and robust first- and second-order features only (model 4). For model 4, the number of robust features included in the model, corresponding to features presenting ICCs and CCCs > 0.8 based on the DATASET1 experiment, are indicated in square brackets. In all configurations, model 3 reached a balanced accuracy similar to that of the best model previously obtained using first-order features only or second-order features only, i.e., model 1 for the T1w-gd sequence, except when a configuration including no normalization with FBN was considered, and model 2 for the T2w-flair sequence. Model 4 resulted in the same classification performances as model 3 in terms of balanced accuracy for the T1w-gd sequence, except when no normalization was coupled to FBS discretization. As an example, considering the T1w-gd sequence, Z-Score normalization, and FBN discretization, the mean classification accuracy was equal to 0.82 (95% CI 0.80–0.86) for model 3 and 0.81 (95% CI 0.78–0.84) for model 4. For the T2w-flair sequence, the accuracy decreased regardless of the considered configuration after applying feature selection. All trends were similar when the number of bins was modified (results not shown).

**Table 4:** Summary of the average balanced accuracies and the corresponding 95% CI (DATASET2) obtained using all 5 test folds of the cross-validation of the 5 machine learning models (neural network, random forest, support vector machine, logistic regression, naïve Bayes) as a function of the normalization method. For both intensity discretization methods (FBN and FBS), 32 bins were used. For model 4, numbers of robust features as defined using DATASET1 are written in square brackets. *BAC* balanced accuracy, *ROC-AUC* area under the receiver operating characteristic curve.

		T1w-gd								T2w-flair							
		Model 1		Model 2		Model 3		Model 4		Model 1		Model 2		Model 3		Model 4	
		BAC	ROC-AUC	BAC	ROC-AUC	BAC	ROC-AUC	BAC	ROC-AUC	BAC	ROC-AUC	BAC	ROC-AUC	BAC	ROC-AUC	BAC	ROC-AUC
FBN	No normalization	0.67 (0.61–0.73)	0.74 (0.68–0.80)	0.80 (0.76–0.83)	0.86 (0.82–0.89)	0.76 (0.71–0.81)	0.83 (0.77–0.88)	0.73 (0.70–0.77) [23]	0.83 (0.80–0.86) [23]	0.62 (0.59–0.64)	0.64 (0.60–0.68)	0.65 (0.62–0.68)	0.70 (0.67–0.73)	0.63 (0.60–0.65)	0.70 (0.66–0.74)	0.60 (0.57–0.63) [23]	0.66 (0.63–0.70) [23]
	Nyul	0.82 (0.79–0.84)	0.90 (0.87–0.92)	0.76 (0.72–0.79)	0.83 (0.80–0.86)	0.81 (0.77–0.84)	0.88 (0.86–0.91)	0.81 (0.78–0.84) [43]	0.89 (0.86–0.92) [43]	0.56 (0.52–0.59)	0.61 (0.58–0.65)	0.67 (0.64–0.69)	0.72 (0.70–0.74)	0.66 (0.64–0.69)	0.71 (0.69–0.74)	0.62 (0.59–0.66) [27]	0.67 (0.63–0.70) [27]
	WhiteStripe	0.79 (0.77–0.82)	0.88 (0.86–0.90)	0.80 (0.76–0.83)	0.86 (0.83–0.90)	0.80 (0.77–0.84)	0.89 (0.86–0.92)	0.79 (0.76–0.83) [28]	0.89 (0.87–0.91) [28]	0.57 (0.54–0.60)	0.63 (0.60–0.67)	0.65 (0.62–0.68)	0.70 (0.67–0.73)	0.65 (0.62–0.67)	0.70 (0.67–0.73)	0.62 (0.59–0.65) [24]	0.67 (0.64–0.71) [24]
	Z-Score	0.82 (0.80–0.85)	0.91 (0.89–0.93)	0.80 (0.76–0.83)	0.86 (0.83–0.90)	0.82 (0.80–0.86)	0.90 (0.88–0.93)	0.81 (0.78–0.84) [32]	0.91 (0.89–0.94) [32]	0.60 (0.57–0.63)	0.65 (0.62–0.69)	0.65 (0.62–0.68)	0.70 (0.66–0.73)	0.67 (0.64–0.70)	0.72 (0.69–0.75)	0.63 (0.60–0.66) [24]	0.68 (0.65–0.72) [24]
FBS	No normalization	0.67 (0.61–0.73)	0.74 (0.68–0.80)	0.68 (0.62–0.72)	0.75 (0.70–0.79)	0.69 (0.63–0.74)	0.75 (0.68–0.81)	0.58 (0.54–0.61) [9]	0.64 (0.59–0.69) [23]	0.62 (0.59–0.64)	0.64 (0.60–0.68)	0.60 (0.58–0.63)	0.64 (0.61–0.68)	0.59 (0.56–0.62)	0.64 (0.60–0.67)	0.56 (0.54–0.59) [7]	0.61 (0.58–0.65) [7]
	Nyul	0.82 (0.79–0.84)	0.90 (0.87–0.92)	0.76 (0.74–0.79)	0.83 (0.80–0.86)	0.81 (0.78–0.84)	0.88 (0.85–0.91)	0.82 (0.79–0.85) [40]	0.89 (0.86–0.91) [43]	0.56 (0.52–0.59)	0.61 (0.58–0.65)	0.64 (0.60–0.68)	0.71 (0.67–0.75)	0.62 (0.59–0.66)	0.70 (0.66–0.73)	0.59 (0.55–0.62) [50]	0.64 (0.61–0.68) [50]
	WhiteStripe	0.79 (0.77–0.82)	0.88 (0.86–0.90)	0.76 (0.72–0.79)	0.84 (0.81–0.87)	0.79 (0.76–0.82)	0.87 (0.84–0.90)	0.79 (0.76–0.82) [20]	0.88 (0.86–0.90) [28]	0.57 (0.54–0.60)	0.63 (0.60–0.67)	0.63 (0.60–0.66)	0.69 (0.67–0.73)	0.61 (0.58–0.64)	0.69 (0.65–0.72)	0.61 (0.58–0.64) [36]	0.68 (0.65–0.71) [36]
	Z-Score	0.82 (0.80–0.85)	0.91 (0.89–0.93)	0.78 (0.75–0.82)	0.86 (0.83–0.89)	0.80 (0.77–0.83)	0.90 (0.87–0.93)	0.83 (0.80–0.85) [45]	0.91 (0.88–0.93) [32]	0.60 (0.57–0.63)	0.65 (0.62–0.69)	0.64 (0.61–0.67)	0.70 (0.67–0.73)	0.64 (0.60–0.67)	0.71 (0.68–0.74)	0.61 (0.58–0.63) [36]	0.66 (0.62–0.69) [36]

The results summarizing the average balanced accuracy and the corresponding 95% CI obtained using all 5 test folds of the cross-validation of the 5 machine learning models as a function of the normalization method and of the number of bins for models 3 and 4 are available in Figures S1 and S2.

To illustrate the robustness of the observations independently of the performance metric, the results corresponding to the ROC-AUC metric for Figs. 1, 3, S1 and S2 are plotted in Figures S3, S4, S5 and S6, respectively.

## Discussion

Radiomics relies on the extraction of features from multimodal imaging, aiming to improve patient care. Although acquisition parameters strongly affect the content of MR images, only some recent studies have specifically focused on the impact of MRI pre-processing methods on radiomics features^10,40,41^. Here, we investigated the impact of three different intensity normalization approaches combined with two grey-level discretization methods on brain MR-based radiomics. In a majority of studies, FBS has, in fact, been presented as the default discretization method based on published PET/CT results^35,42^. This conclusion is relevant for quantitative or semi-quantitative modalities (e.g., HU in CT, SUV in PET) for which intensities have a physical meaning. In MRI, intensity values strongly depend on acquisition parameters, making the generalization of radiomics models even more challenging^38^. Recently, the IBSI has proposed recommendations for each imaging modality^43^. For MRI, a relative discretization is recommended to account for the variable intensity ranges.

First, we demonstrated that the use of an intensity normalization step improves the robustness of the first-order and FBS-based textural features using DATASET1 (Table 3 and Fig. 2C) and associated performances on the classification task based on T1w-gd images (Figs. 1A, 3C—DATASET2). Nyul’s harmonization method, based on a reference histogram, leads to the highest number of robust first-order features (Table 3). However, it has already been shown that this piecewise linear transformation affects the texture of the images^33^. Additionally, piecewise mapping can be distorted when large tumours are considered. These observations are in accordance with our results showing that different texture feature values were obtained with the Nyul method compared to no normalization and the WhiteStripe and Z-Score methods with FBN discretization (Fig. 2A,B). WhiteStripe intensity normalization performs a Z-Score normalization based on NAWM values. The WhiteStripe method is dependent on the accuracy of the white matter segmentation, which can affect the quality of the normalization. In contrast, the Z-Score method is the simplest to implement, requires only a short computation time and is the most robust method because it considers all the voxels inside the brain mask. This latter produces very good results in terms of classification performances (Figs. 1, 3, S1 and S2) independent of the MR sequence and the grey-level discretization method, even though no statistical significance was achieved. Overall, normalization has a greater positive impact on the T1w-gd sequence than on the T2w-flair sequence. This is mainly because the intensity range of raw MR images is, on average, 5 times lower on T2w-flair images than on T1w-gd images. With the additional use of a grey-level discretization step for textural feature computation, intensity normalization is mandatory when absolute discretization is preferred for T1w-gd images (Fig. 3C). Classification performances obtained on DATASET2 highlight that intensity normalization is not needed when relative discretization is applied, making the pre-processing steps of skull stripping and intensity normalization unnecessary (Fig. 3A,B).

The evaluation of the impact of the number of bins for discretization is not trivial. Even if high numbers of bins increase feature robustness in the majority of the cases (Fig. 2), they tend to decrease performance in terms of classification accuracy when considering the T2-flair sequence (Fig. 3B). Goya-Outi et al*.* investigated the impact of intensity binning combined with WhiteStripe normalization on 30 patients suffering from diffuse intrinsic pontine glioma^44^. They compared patient ranking based on radiomics features to visual assessment of the heterogeneity. The dataset was obtained using a single MR device and included 4 MR sequences (T1w, T1w-gd, T2w and T2w-flair). Three types of intensity binning were compared: (1) a constant bin size and relative bounds (FBS); (2) a constant number of bins and relative bounds (FBN); and (3) a constant number of bins and absolute bounds. For 20 out of the 240 indices, patient rankings obtained with binning (1) and (2) were highly correlated (|r|> 0.7). This number increased to 188 when comparing rankings obtained with binning (2) and (3) and was reduced to 9 when comparing (2) and (3). They subsequently adopted the absolute discretization (1), as it does not require the setting of absolute lower and upper bounds. Goya-Outi et al*.* have shown similar patient rankings for the large majority of 240 textural features when using different values of FBN (8, 16, 32, 64, 128) or FBS (0.75, 1, 2, 3, 4). More recently, Duron et al*.* evaluated the influence of grey-level discretization on inter- and intra-observer reproducibilities of textural features extracted from 6 MR sequences^39^ based on manual and automatic segmentations. FBS was shown to be associated with a higher number of reproducible features based on a combination of ICCs and CCCs. In this study, the authors did not normalize the intensities before feature extraction, but they also did not limit the conclusions to a selected range of bin sizes or numbers. In our study, we found that the choice of the number of bins leads to small differences between 16 and 128 bins, with a maximum variation of 10% in the percentages of robust features (Fig. 2—DATASET1) regardless of the sequence and normalization method. Regarding the classification, increasing the number of bins above 128 significantly reduced the accuracy of the classification for the T2w-flair sequence for the FBN discretization. Based on our results (Figs. 3, S1 and S2), a number of bins equal to 32 seems to be a good compromise for brain MR analysis after Z-Score normalization, as it leads to the most informative radiomics signatures for both sequences, with acceptable calculation times.

Preliminary feature selection based on robustness is widely used in radiomics^45,46^. In the present study, no improvements in classification performances were observed using feature selection (Figure S2, Table 4). These results suggest that, considering brain MR data for a grade classification task, a step of feature selection based on feature robustness could be optional.

Most recently, 2 publications focused on the image pre-processing steps and their impact on radiomics feature reproducibility in brain patients. Moradmand et al.^10^ evaluated the impact of 5 combinations of image pre-processing on the reproducibility of 1461 radiomic features (i.e., spatial resampling, skull stripping, noise reduction, bias field correction and intensity normalization) extracted from different glioblastoma (GBM) subregions (i.e., oedema, necrosis, enhanced tumour). They showed that radiomics features extracted from necrotic regions were the most reproducible and recommended that, after the bias field correction step, noise filtering should be applied. In that work, no analysis of the optimal pre-processing based on a clinical classification or regression task was performed, making it difficult to compare their results to ours. In 2019, Um et al.^40^ studied the impact of image pre-processing methods on 420 radiomics features extracted from MR images from two datasets: 50 patients from the TCGA-GBM dataset and 111 institutional patients. They evaluated five image pre-processing techniques: 8-bit global rescaling, 8-bit local rescaling, bias field correction, histogram normalization and isotropic resampling. Their goal was to evaluate the ability of a machine learning classifier to classify each patient according the cohort to which a patient belongs (covariate shift) depending on the pre-processing step performed. They also assessed the impact of each pre-processing step on an overall survival model. They showed that no single pre-processing step was sufficient to completely remove the machine effect. However, in their cohort, histogram normalization combined with a relative grey-level discretization (16, 32, 64 and 128 bins) was the most important step in reducing inter-machine effects. Compared to our study, they did not analyse the impact of different methods of normalization or discretization. Moreover, the comparison of their results to ours is difficult, as no interplay effect of the different pre-processing methods was analysed. In addition, there was no use of "skull stripping" prior to the application of intensity rescaling, which should have been a mandatory step^47^. Finally, this comparison is also challenging due to different cohorts and tasks applied.

Additional studies are awaited to confirm our results, which also need to be validated in other tasks. Of note, cross-validation was used to assess classification performances. Even if the use of an independent test set would have been preferable, the various train-test partitions combined with a bootstrapping strategy allowed us to draw conclusions efficiently. Regularization methods will have to be implemented in future studies to decrease the risk of overfitting. In addition, only anatomical MR sequences have been considered. These images are, however, the conventional sequences for radiological assessment of cerebral lesions; the use of more quantitative functional imaging is still sparse in clinical practice. In this study, a unique ROI was delineated; thus, the choice of the ideal number of bins can be influenced by the sharpness of the intensities at the border of the lesion. As the number of voxels included in the tumour was negligible compared to the number of voxels in the whole brain (i.e., the volume of the tumour was equal to 7.5 ± 3.7% of the whole brain in DATASET2), no tumour exclusion was applied during the normalization process. This assumption could have biased, to a limited extent, the implementation of the normalization algorithms. In the second experiment, in which a classification task was studied, the results from DATASET1 regarding feature reproducibility were considered for feature selection in model 4. In DATASET1, a narrow set of acquisition and reconstruction parameters was investigated and compared to real-life disparity, emphasizing the need for additional studies. Finally, some pre-processing step parameters, such as bias field correction and spatial resampling, could have affected comparisons. These two pre-processing methods have still been used in a large number of published studies that have demonstrated their importance for the robustness of features^48–50^. Recently, a compensation method to pool radiomics features from different centres has been suggested. This data-driven post-processing method, called ComBat^51^, seems to be able to harmonize radiomics data a posteriori. Initially proposed to correct batch effects in genomic studies, ComBat has demonstrated its effectiveness in PET^52^ and CT^53^. The next step will consist of comparing ComBat with the pre-processing methods described in this article.

In conclusion, a standardized pre-processing pipeline is recommended for brain tumour radiomics analyses. For models based on first- and second-order features, the combination of Z-Score normalization and absolute discretization seems to be the best of the methods tested. For works that consider only second-order features, the relative discretization without prior intensity normalization seems to be sufficient. Even if the bin number for the discretization has a small impact on classification performances, 32 bins appear to be a good compromise when T1w-gd and T2w-flair sequences are considered. The pre-processing methods used must be described in detail in the published papers to achieve reliable radiomics-based machine learning models. Such a pipeline will be pivotal for the implementation of large-scale multicentric studies and may pave the way for the development and validation of MR-based radiomics biomarkers.

## Material and methods

### Data description

Two retrospective datasets were used for this study. DATASET1 included twenty consecutive patients with WHO grade II and III gliomas between January and June 2010 (Table 5). A previous article based on the same cohort analysed the robustness of conventional features (lesion volumes, ratios of cerebral blood volumes, contrast-to-noise ratios) depending on the magnetic field^54^. In this manuscript, the same cohort was considered to evaluate the stability of first-order and second-order radiomics features across acquisitions. Each patient underwent two MR acquisitions on 1.5 T (Signa EchoSpeed, GE Healthcare, Milwaukee, Wisconsin, USA) and 3 T (Discovery MR750, GE Healthcare) scanners, with a mean interval of 7.4 (± 3.0) days. Inclusion criteria supposed that no clinical or morphological change related to the glioma occurred during this delay. This was certified by a blinded radiologist (SA, 10 years of experience, with 5 years of specialization in neuro-oncology). A post-contrast 3D axial T1-weighted (T1w-gd) sequence and an axial T2-weighted fluid attenuation inversion recovery (T2w-flair) sequence were acquired on each scanner.

**Table 5:** Datasets description including MR acquisition parameters.

Parameters	DATASET1				DATASET2^a^			
Sequence	T1w-gd		T2w-flair		T1w-gd		T2w-flair	
Manufacturer model	GE Signa HDxt	GE Discovery MR750	GE Signa HDxt	GE Discovery MR750	Philips AchievaSiemens (17) GE Signa Genesis (52) GE Signa Excite (71) GE Signa HDx (3) GE Signa HDxt (8) Siemens Magnetom Vision (10) Hitachi Oasis (1) Philips Ingenia (6) Philips Intera (6) Philips Intera Achieva (1) Siemens Avanto (9) Siemens Skyra (1) Siemens Symphony (10) Siemens Trio (2) Siemens TrioTim (3) Siemens Verio (5) Undefined (38)			
Cohort					LGG	HGG	LGG	HGG
Magnetic field strength (T)	1.5	3.0	1.5	3.0	1.16 (N = 1), 1.5 (N = 51), 3.0 (N = 47), undefined (N = 9)	0.5 (N = 2), 1 (N = 1), 1.5 (N = 82), 3.0 (N = 44) undefined (N = 6)	1.16 (N = 1), 1.5 (N = 51), 3.0 (N = 47), undefined (N = 9)	0.5 (N = 2), 1 (N = 1), 1.5 (N = 82), 3.0 (N = 44) undefined (N = 6)
TR (ms)	11	10	9802	8000	1106 [6–5500]	890 [5–3286]	9686 [6000–11,000]	9581 [1000–11,000]
TE (ms)	4	3	157	123	7 [3–17]	9 [2–105]	128 [94–158]	135 [74–355]
Slice thickness (mm)	1.4	1.2	5.0	3.5	2.4 [1.0–5.0]	3.2 [1.0–6.0]	3.8 [2.0–5.0]	4.14 [1.2–6.0]
Pixel spacing (mm)	0.49 × 0.49	0.47 × 0.47	0.47 × 0.47	0.43 × 0.43	0.68 × 0.68 [0.39 × 0.39–1.02 × 1.02]	0.77 × 0.77 [0.43 × 0.43–1.02 × 1.02]	0.74 × 0.74 [0.39 × 0.39–1.01 × 1.01]	0.77 × 0.77 [0.43 × 0.43–1.01 × 1.01]
Matrix dimensions	288 × 288	320 × 288	256 × 192	352 × 192	303 × 2130 [224 × 134–512 × 300]	283 × 204 [224 × 134–512 × 300]	306 × 214 [256 × 112–512 × 256]	283 × 194 [192 × 98–512 × 320]
FOV (mm)	250	240	240	220	244 [200–260]	235 [200–260]	237 [200–260]	228 [200–260]
Pixel bandwidth (Hz/px)	65.12	65.12	122	195	166 [81–250]	162 [61–355]	153 [61–358]	170 [61–750]
Flip angle (°)	17	15	90	90	53 [8–90]	70 [8–90]	100 [90–180]	102 [90–180]

*TR* repetition time, *TE* echo time, *FOV* field of view.

^a^Some metadata information are missing (< 10% of all patients). For the DATASET2, values representations are: mean [min–max]. The number of patients for each MR system is indicated in brackets. Additional information about DATASET2 are available in Bakas et al. ^34,35^.

DATASET2 included pre-operative multi-institutional scans of The Cancer Genome Atlas (TCGA) Glioblastoma Multiforme (GBM) and Low-Grade Glioma (LGG) collections, publicly available in The Cancer Imaging Archive (TCIA). A total of 135 and 108 exams, including T1w-gd and T2w-flair sequences extracted from the TCGA-GBM and TCGA-LGG cohorts, respectively, were used (Table 5)^55–57^.

### Image pre-processing

MR images from DATASET1 and DATASET2 were first corrected for the bias field effect using the N4ITK algorithm^49^ as implemented in the Advanced Normalization Tools (ANTs)^58^ with default parameters. They were then spatially resampled on a 1 mm × 1 mm × 1 mm grid as suggested by Vallières et al.^50^ using b-spline interpolation with ANTs. Images from DATASET1 were finally skull-stripped with the Brain Extraction Tool (BET) of the FSL software (FMRIB's Software Library)^59^ and co-registered with a global linear registration including 12 degrees of freedom using ANTs to the T1w-gd sequence, considered as the reference. As some differences occurred in the skull stripping between the 1.5 T and 3 T images for the same MR sequence, an intersection between the two masks was performed. For DATASET2, the method described by Bakas et al*.* was used for co-registration to recover the spatial domain in which the segmentations were performed^55,60^. Brain masks provided by Bakas et al*.* were applied for skull stripping. In both cases, MR images were finally normalized using 3 different methods (Nyul, WhiteStripe, Z-Score).

The Z-Score method normalizes image histograms by subtracting $(\mu_{brain} )$, corresponding to the mean intensity value of the considered ROI (here, the brain), from each voxel intensity I(x) and dividing the result by the standard deviation of the ROI $(\sigma_{brain} )$:

1 $$ I_{{Z{\text{-}}Score}} (x) = \frac{{I(x) - \mu_{brain} }}{{\sigma_{brain} }} $$

The WhiteStripe method normalizes image intensities by subtracting $(\mu_{ws} )$, which corresponds to the mean intensity value of the normal-appearing white matter (NAWM), from each voxel intensity I(x) and dividing the result by the standard deviation of the NAWM $(\sigma_{ws} )$^33^. As conventionally applied in the literature, the “white stripe” region was defined automatically in this work, using a threshold in intensities, corresponding to ± 5% of $(\mu_{ws} )$.

2 $$ I_{WhiteStripe} (x) = \frac{{I(x) - \mu_{ws} }}{{\sigma_{ws} }} $$

Nyul’s method corresponds to piecewise linear histogram matching^31^. The normalization problem is addressed by learning a standard histogram from a set of images and linearly mapping the intensities of each image of interest to this standard histogram. The standard histogram is learned by averaging predefined landmarks deduced from histograms of the training set. The intensity landmark configuration $C_{L} = \left[ {1,10,20,30,40,50,60,70,80,90,99} \right]$ (intensity percentiles) chosen in this study corresponds to the one defined by Shah et al.^32^.

Note that for the normalization process, no tumour exclusion from the brain mask was applied.

More details about intensity normalization methods can be found in the original papers^31–33^. The code used in this paper as well as details about the algorithm implementation^61^ are available at https://github.com/jcreinhold/intensity-normalization.

### Segmentation

A unique ROI including the tumour and peritumoral oedema was considered. These ROIs were segmented for DATASET1 by an experienced radiation oncologist (GK, 4 years of experience) using the 3D Slicer open-source platform version 4.10.1 (https://www.slicer.org). For DATASET2, the labelled regions supplied by Bakas et al*.* were merged.

### Feature extraction and grey-level discretization

The open-source Pyradiomics package (version 2.1.2) was used to extract 18 first-order statistics and 73 textural features from the segmented tumour regions of both DATASETS^62^. The 5 texture feature classes were based on the grey-level co-occurrence matrix (GLCM, 22 features), grey-level run length matrix (GLRLM, 16 features), grey-level size zone matrix (GLSZM, 16 features), neighbourhood grey tone difference matrix (NGTDM, 5 features) and grey-level dependence matrix (GLDM, 14 features). Except for 4, all the features conformed to the definition provided by the Imaging Biomarker Normalization Initiative (IBSI)^43^. All the features used in this study are listed in Supplementary Data S7.

To assess the impact of the intensity discretization method on textural features, two approaches of grey-level discretization commonly used in the literature were implemented.

The FBS method assigns the same bin for every voxel intensity corresponding to the bin width $w_{b}$. It is defined as follows:

3 $$ X_{d,k} = \left\lfloor {\frac{{X_{gl,k} }}{{w_{b} }}} \right\rfloor - \left\lfloor {\frac{{X_{gl,min} }}{{w_{b} }}} \right\rfloor + 1 $$

where the minimum intensity in the ROI, $X_{gl,min}$, is subtracted from intensity $X_{gl,k}$, corresponding to the intensity of voxel $k$, and divided by the bin width $w_{b}$. $\left\lfloor {\frac{{X_{gl,min} }}{{w_{b} }}} \right\rfloor + 1$ ensures that the grey-level rebinning starts at 1.

The FBN method discretizes every voxel intensity from an ROI to a fixed number of $N_{g}$ bins. It is defined as follows:

4 $$ X_{d,k} = \left\{ {\begin{array}{*{20}l} {\left\lfloor {N_{g} \frac{{X_{gl,k} - X_{gl, min} }}{{X_{gl, max} - X_{gl, min} }}} \right\rfloor + 1,} \hfill & {X_{gl,k} < X_{gl, max} } \hfill \\ {N_{g} ,} \hfill & {X_{gl,k} = X_{gl, max} } \hfill \\ \end{array} } \right. $$

where $N_{g}$ corresponds to the fixed number of bins between $X_{gl,min}$ and $X_{gl,max}$, which are the minimum and maximum intensities of the ROI, respectively.

To correctly analyse the impact of grey-level discretization on pre-processed images on which the intensity ranges can be different, a scaling factor was computed for the FBS method, as shown in Eq. (5):

5 $$ FBS = \frac{1}{FBN} \times mean_{Range} $$

where $mean_{Range}$ corresponds to the mean of the intensity intervals computed for all patient ROIs for one MR sequence. For the two datasets, 8 different bin numbers were applied: 8, 16, 32, 64, 128, 256, 512 and 1024.

### Data analysis

R software (version 3.6.0) was used for the statistical analysis. Regarding DATASET1, JSD was used to compare each pair of intensity histograms before and after normalization^63^. A one-way analysis of variance (ANOVA) test was conducted to compare JSD values among the normalization methods. If the ANOVA test was statistically significant, a subsequent pairwise post hoc Tukey’s multiple comparison test was performed. For both tests, a *P *value < 0.05 was considered significant. The CCCs and ICCs were computed to assess the stability of first-order and textural features across the two acquisitions before and after normalization (Supplementary Data S8). There are currently no conclusions on the optimal thresholds to be used for ICCs and CCCs. In the literature, the most commonly used values are 0.8 for the ICC and 0.85 to 0.9 for the CCC^45,64^. Lecler et al.^45^ showed in 2019 that a CCC threshold of 0.9 overrides the value imposed by the ICC. Thus, it was concluded that a too-restrictive threshold could lead to loss of valuable information. In this work, radiomics features were defined as robust if the ICC and the CCC were > 0.8.

DATASET2 aimed to evaluate the usefulness of intensity normalization and to define the optimal grey-level discretization for a tumour grade classification task. Five widely used classifiers were implemented based on the scikit-learn library version 0.20.3^65^. These included random forest, naïve Bayes, logistic regression, support vector machine and neural network multi-layer perception classifiers. Default parameters were chosen to prevent overfitting. Multiple classifiers were used to avoid limiting the conclusions to a single machine learning model. Moreover, a five-fold stratified cross-validation was adopted*.* In all cases, feature values were normalized using the Z-Score method within the cross-validation. The average values of the balanced accuracies and the ROC-AUC and corresponding 95% CIs evaluated using the five left-out folds of the 5 machine learning models were reported. For the 95% CIs, bootstrapping including 1000 iterations was applied. Balanced accuracy is a performance metric that should be preferred to accuracy in the case of imbalanced datasets^66^. Model 1 included first-order features alone. Model 2 was based on textural features only. The added value of the combination of the two types of features was analysed in model 3. Model 4 included only features defined as robust, i.e., having both an ICC and a CCC > 0.8 in the DATASET1 experiment. A two-way ANOVA test was conducted to simultaneously evaluate the effect of normalization and discretization. If the ANOVA test was statistically significant, a subsequent pairwise post hoc Tukey’s multiple comparison test was performed. For both tests, a *P* value < 0.05 was considered significant.

**Figure 4:**
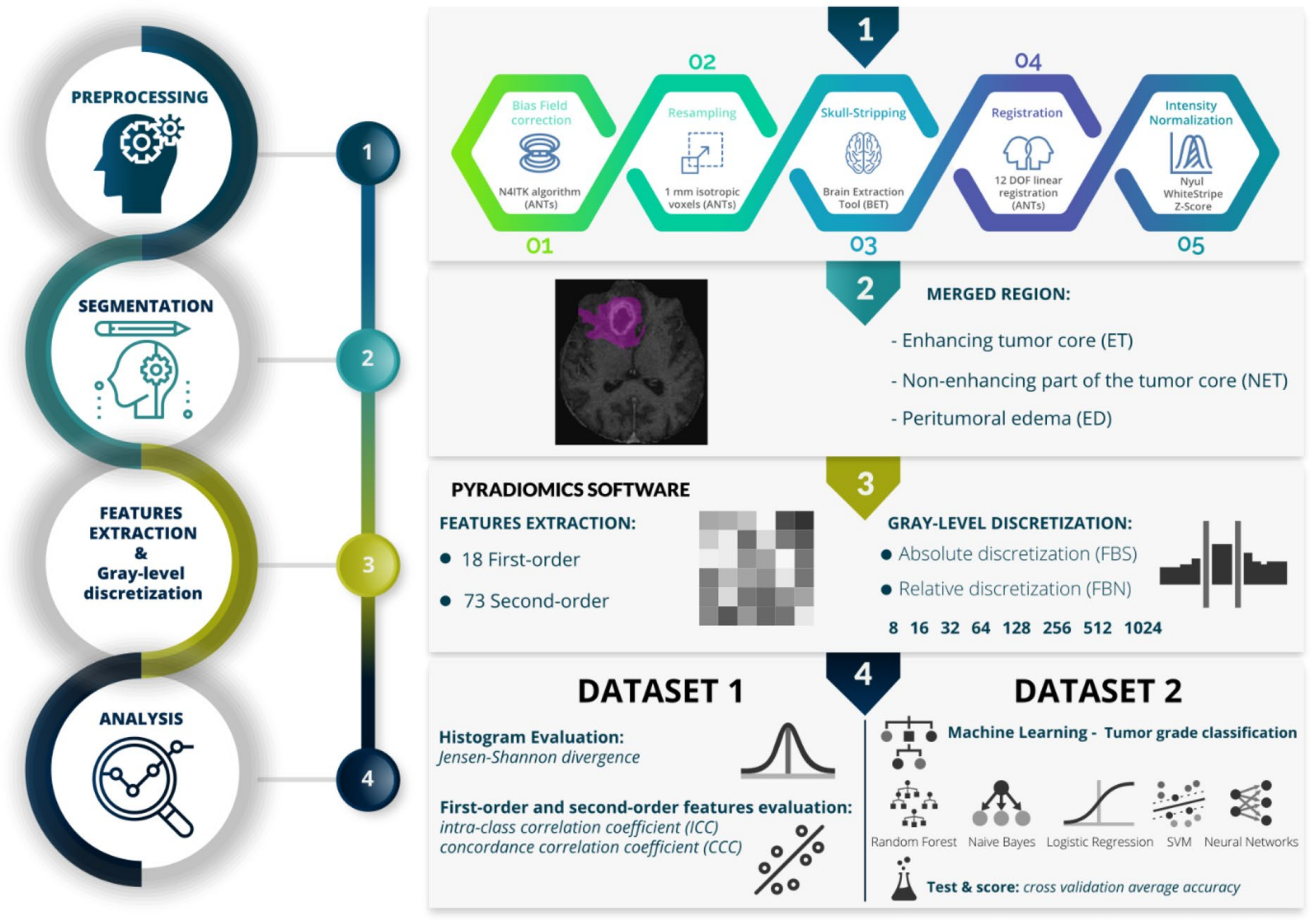
Design of the study.

The design of the study is detailed in Fig. 4.

## Supplementary information

Supplementary information.
