# Supplementary material for: Standardization of brain MR images across machines and protocols: bridging the gap for MRI-based radiomics
Source: Sci Rep. 2020 Jul 23;10:12340. doi: 10.1038/s41598-020-69298-z (PMC7378556; doi:10.1038/s41598-020-69298-z)
Supplement: Supplementary file 1 — Supplementary information. [file 41598_2020_69298_MOESM1_ESM.docx]

Supplementary Material:

TITLE: Standardization of brain MR images across machines and protocols: bridging the gap for MRI-based radiomics

SHORT TITLE: Standardization for brain MRI radiomics

AUTHORS:

Alexandre Carré^1,2^ (Msc), Guillaume Klausner^1,2^ (MD, Msc), Myriam Edjlali^3,4,5^ (MD, PhD), Marvin Lerousseau^1,6^ (Msc), Jade Briend-Diop^1^ (Msc), Roger Sun^1,2,6^ (MD, Msc), Samy Ammari^7^ (MD, Msc), Sylvain Reuzé^1,2^ (PhD), Emilie Alvarez Andres^1,8^ (Msc), Théo Estienne^1,6^ (Msc), Stéphane Niyoteka^1,2^ (Msc), Enzo Battistella^1,6^ (Msc), Maria Vakalopoulou^6^ (PhD), Frédéric Dhermain^2^ (MD, PhD), Nikos Paragios^6,8^ (PhD), Eric Deutsch^1,2^ (MD, PhD), Catherine Oppenheim^3,4,5^ (MD, PhD), Johan Pallud^4,5,9^ (MD, PhD), Charlotte Robert*^1,2^ (PhD)

**S1** Figure.


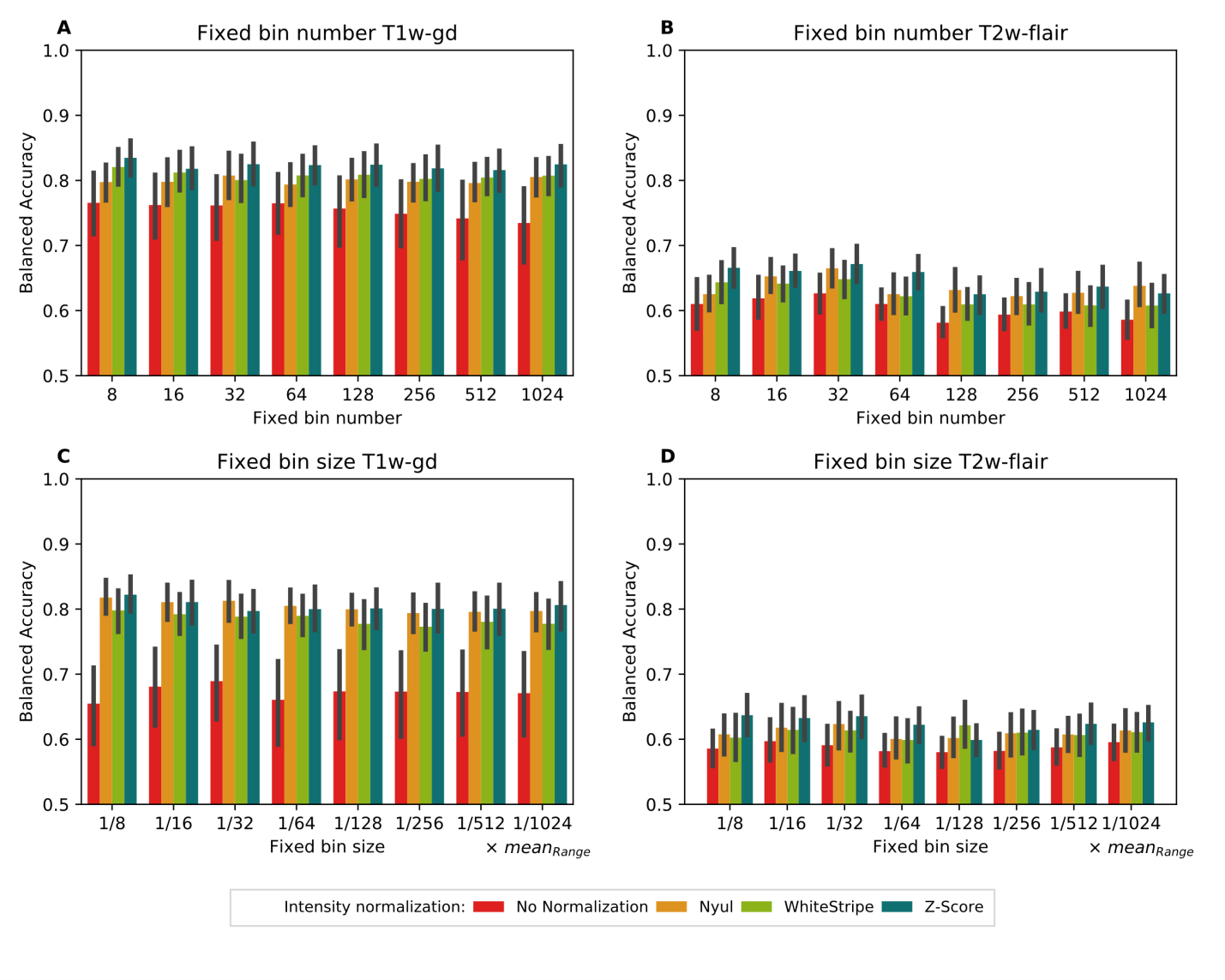


Figure S1: Balanced accuracies obtained for the tumour grade classification task using a combination of the 18 first-order and 73 textural features. Bar plots and associated error bars represent the average balanced accuracies and the 95% CI obtained using all 5 test folds of the cross-validation of the 5 machine learning models as a function of the normalization method and number of bins, respectively. A) FBN T1w-gd. B) FBN T2w-flair. C) FBS T1w-gd. D) FBS T2w-flair. FBN = fixed bin number (relative discretization). FBS = fixed bin size (absolute discretization).

**S2** Figure.

*
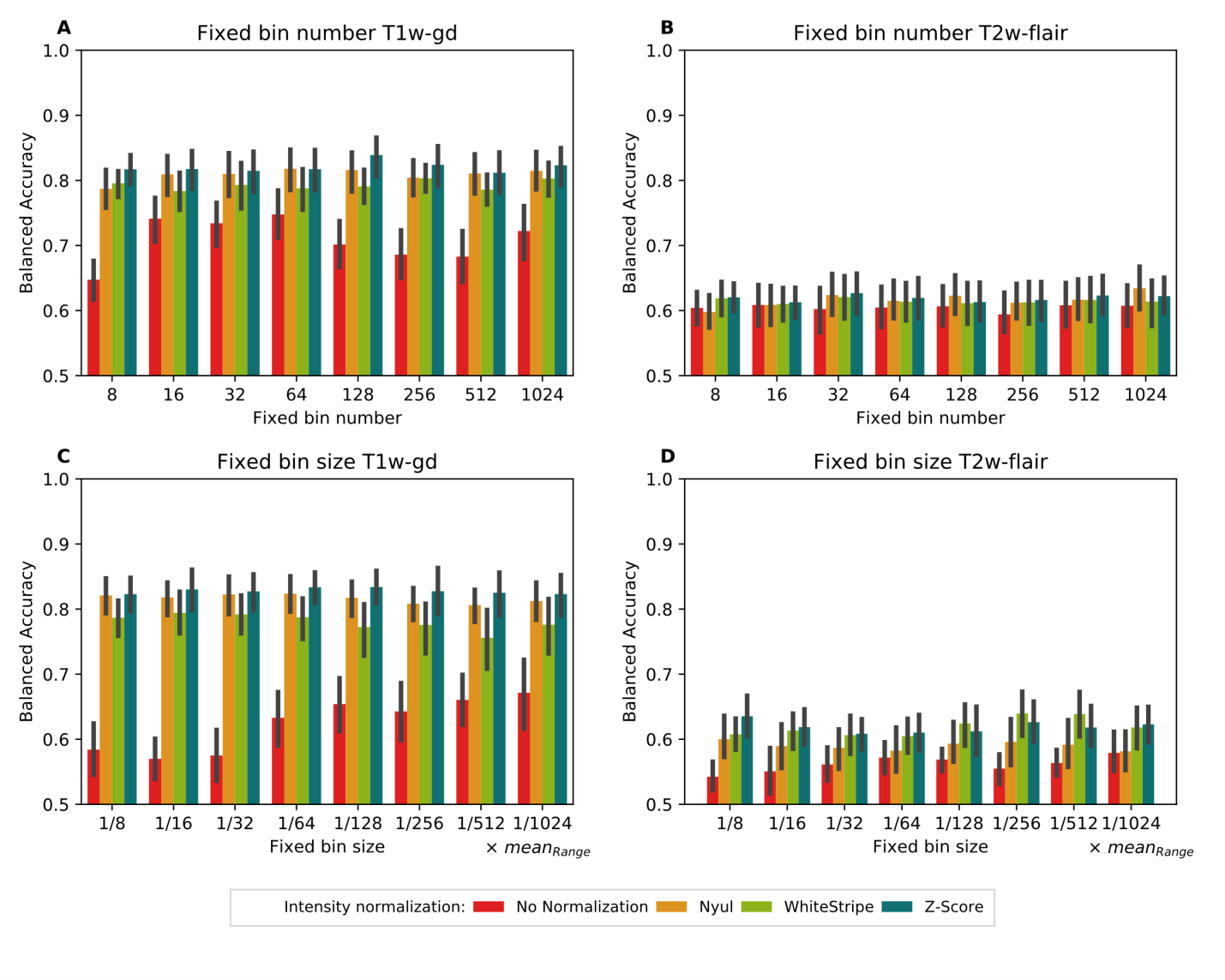
*

Figure S2: Balanced accuracies obtained for the tumour grade classification task using the features defined as robust based on the DATASET1 results. Bar plots and associated error bars represent the average balanced accuracies and the 95% CI obtained using all 5 test folds of the cross-validation of the 5 machine learning models as a function of the normalization method and number of bins, respectively. A) FBN T1w-gd. B) FBN T2w-flair. C) FBS T1w-gd. D) FBS T2w-flair. FBN = fixed bin number (relative discretization). FBS = fixed bin size (absolute discretization).

**S3** Figure.


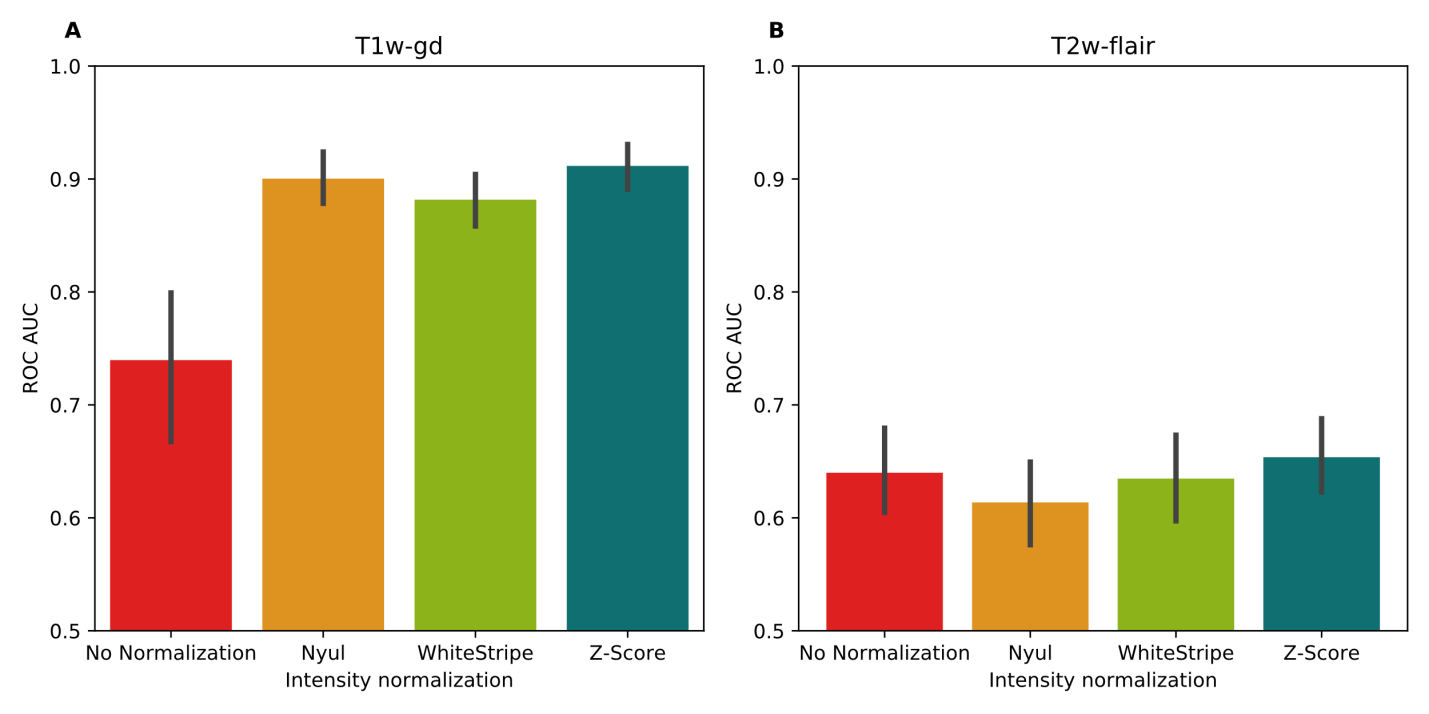


Figure S3: ROC-AUC obtained for the tumour grade classification task using the 18 first-order features only. Bar plots and associated error bars represent the average ROC-AUC and the 95% CI obtained using all 5 test folds of the cross-validation of the 5 machine learning models as a function of the normalization method, respectively. (A) T1w-gd MRI sequence only, (B) T2w-flair MRI sequence only.

**S4** Figure.

*
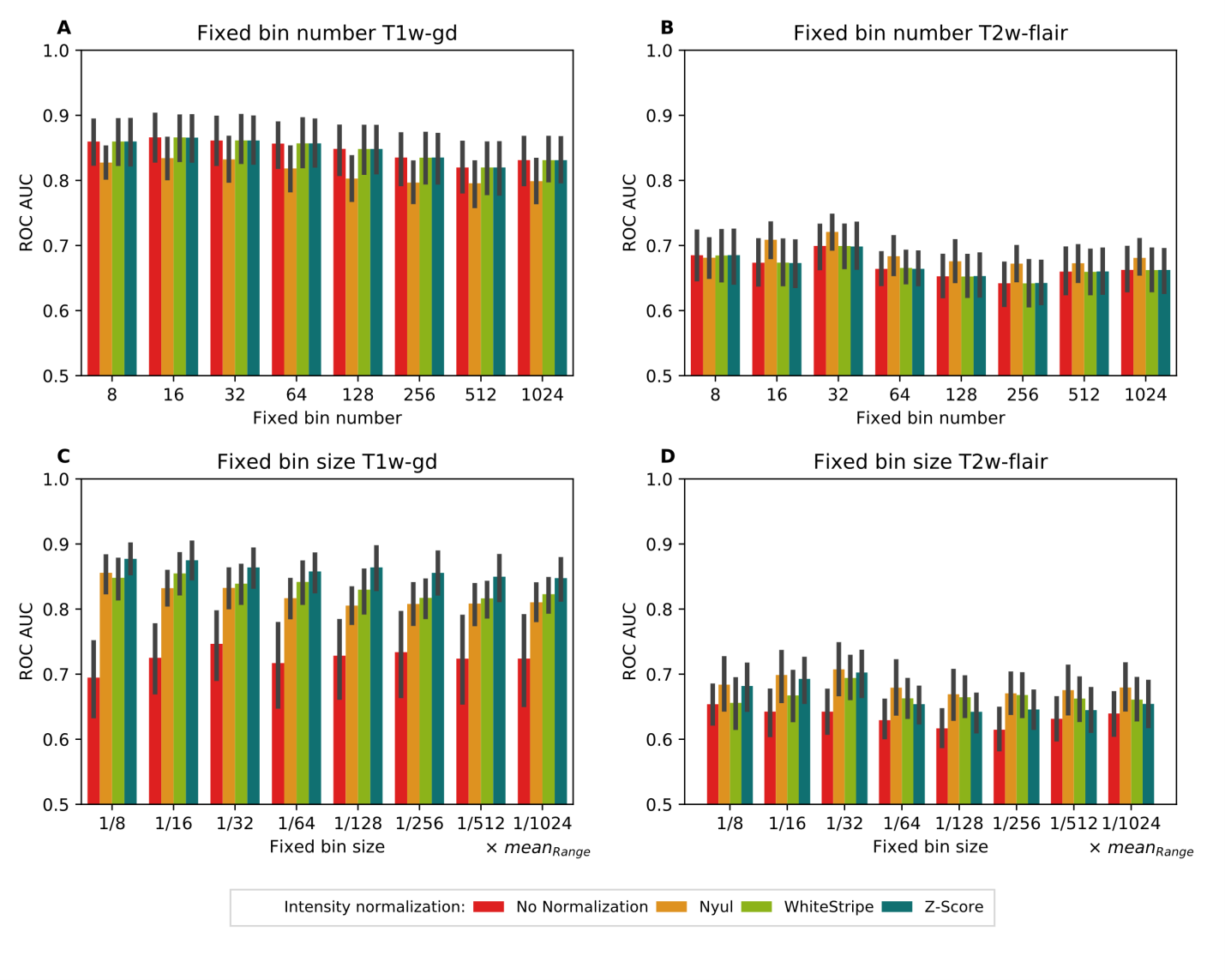
*

Figure S4: ROC-AUC obtained for the tumour grade classification task using the 73 textural features only. Bar plots and associated error bars represent the average ROC-AUC and the 95% CI obtained using all 5 test folds of the cross-validation of the 5 machine learning models as a function of the normalization method and number of bins, respectively. A) FBN T1w-gd. B) FBN T2w-flair. C) FBS T1w-gd. D) FBS T2w-flair. FBN = fixed bin number (relative discretization). FBS = fixed bin size (absolute discretization).

**S5** Figure.

*
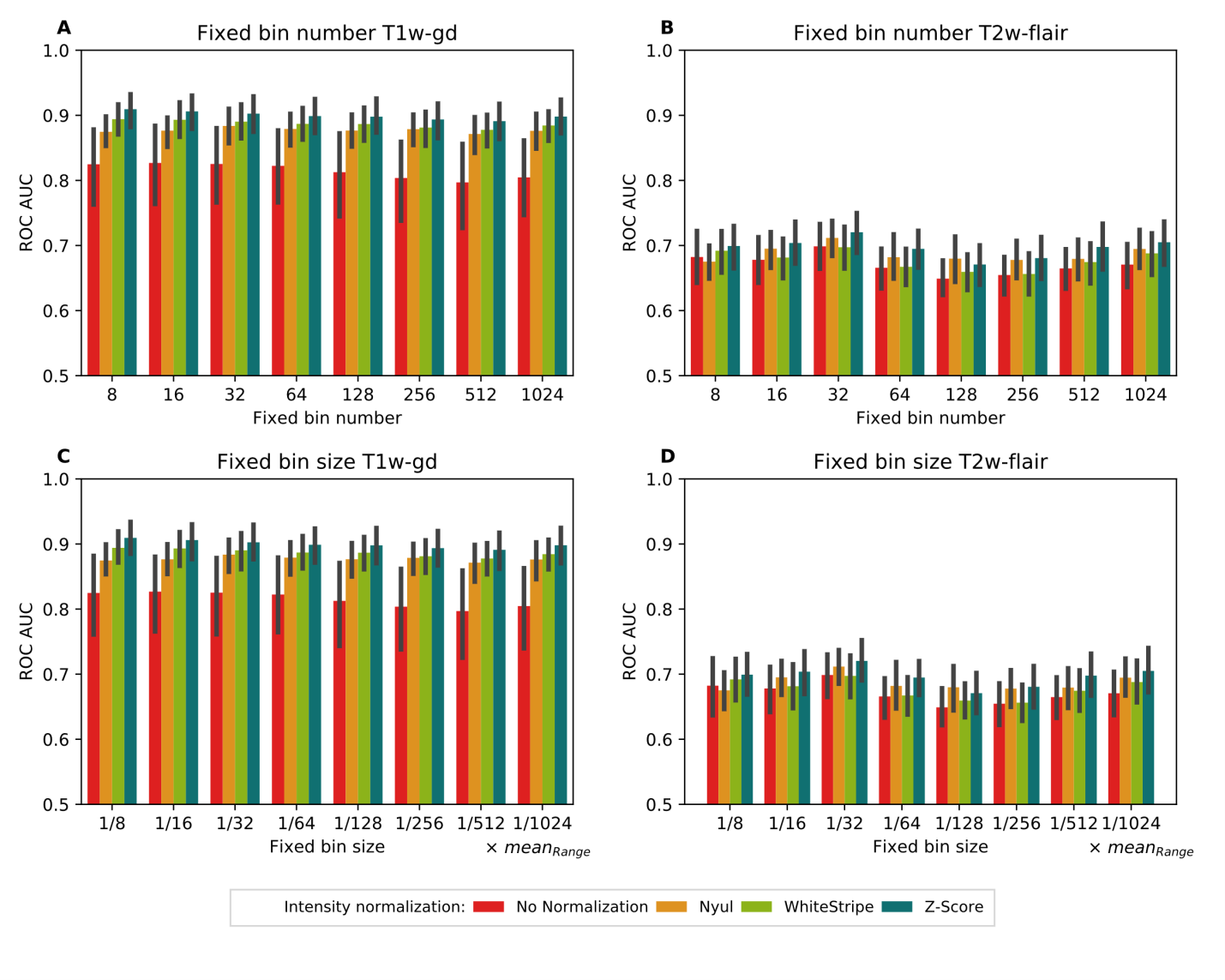
*

Figure S5: ROC-AUC obtained for the tumour grade classification task using a combination of the 18 first-order and 73 textural features. Bar plots and associated error bars represent the average ROC-AUC and the 95% CI obtained using all 5 test folds of the cross-validation of the 5 machine learning models as a function of the normalization method and number of bins, respectively. A) FBN T1w-gd. B) FBN T2w-flair. C) FBS T1w-gd. D) FBS T2w-flair. FBN = fixed bin number (relative discretization). FBS = fixed bin size (absolute discretization).

**S6** Figure.


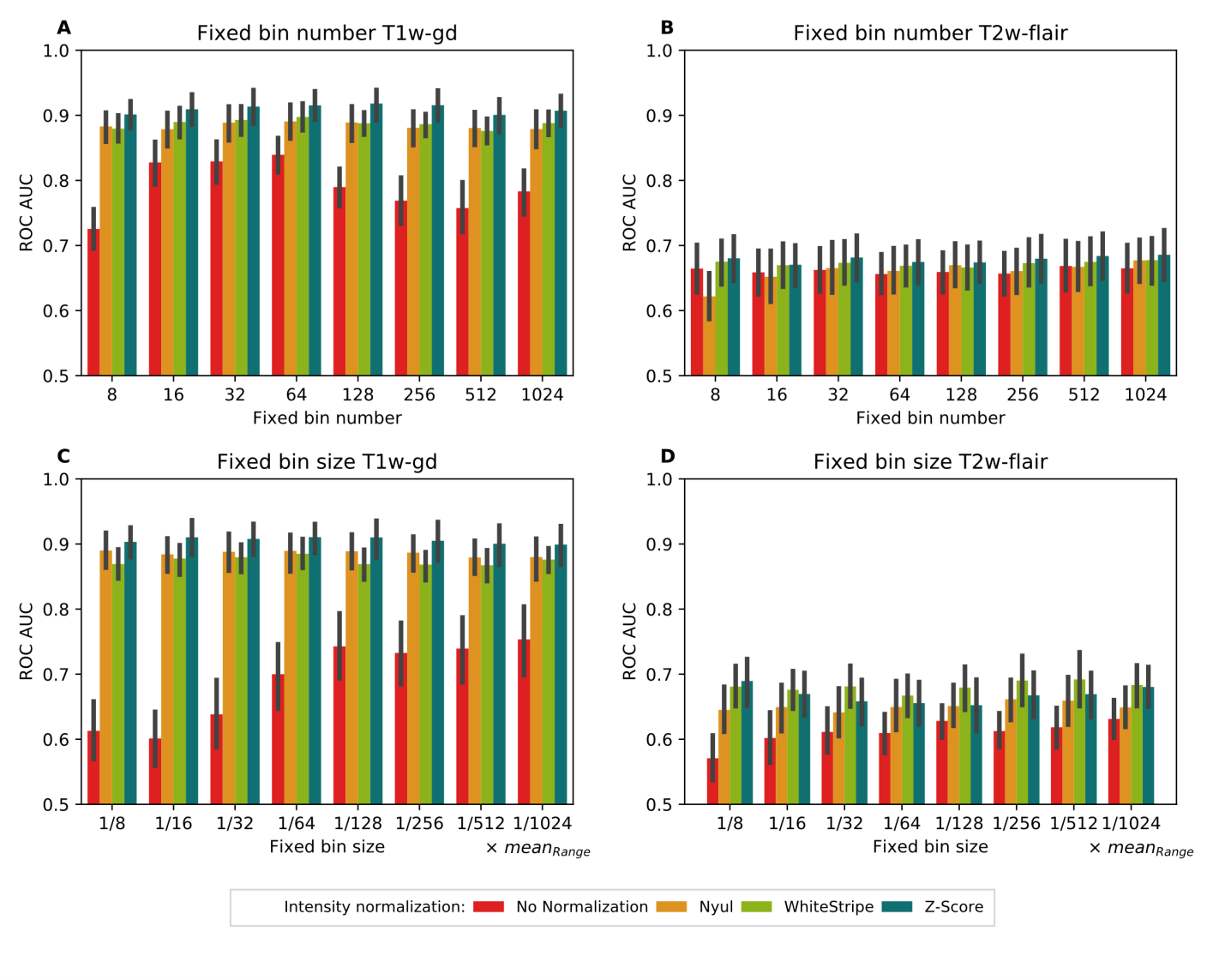


Figure S6: ROC-AUC obtained for the tumour grade classification task using the features defined as robust based on the DATASET1 results. Bar plots and associated error bars represent the average ROC-AUC and the 95% CI obtained using all 5 test folds of the cross-validation of the 5 machine learning models as a function of the normalization method and number of bins, respectively. A) FBN T1w-gd. B) FBN T2w-flair. C) FBS T1w-gd. D) FBS T2w-flair. FBN = fixed bin number (relative discretization). FBS = fixed bin size (absolute discretization).

**S7** Table. List of features considered using Pyradiomics.

| Order of features | Description | Features name |
| --- | --- | --- |
| First | First order statistics | Energy  Total Energy  Entropy  Minimum  10^th^ percentile  90^th^ percentile  Maximum  Mean  Median  Interquartile Range  Range  Mean Absolute Deviation (MAD)  Robust Mean Absolute Deviation (rMAD)  Root Mean Squared (RMS)  Standard Deviation  Skewness  Kurtosis  Variance  Uniformity |
| Second | Grey level co-occurrence matrix (GLCM) | Autocorrelation  Joint Average  Cluster Prominence  Cluster Shade  Cluster Tendency  Contrast  Correlation  Difference Average  Difference Entropy  Difference Variance  Joint Energy  Joint Entropy  Informational Measure of Correlation (IMC) 1  Informational Measure of Correlation (IMC) 2  Inverse Difference Moment (IDM)  Maximal Correlation Coefficient (MCC)  Inverse Difference Moment Normalized (IDMN)  Inverse Difference (ID)  Inverse Difference Normalized (IDN)  Inverse Variance  Maximum Probability  Sum Entropy  Sum of Squares |
| Third | Grey level size zone matrix (GLSZM) | Small Area Emphasis (SAE)  Large Area Emphasis (LAE)  Grey Level Non-Uniformity (GLN)  Grey Level Non-Uniformity Normalized (GLNN)  Size-Zone Non-Uniformity (SZN)  Size-Zone Non-Uniformity Normalized (SZNN)  Zone Percentage (ZP)  Grey Level Variance (GLV)  Zone Variance (ZV)  Zone Entropy (ZE)  Low Grey Level Zone Emphasis (LGLZE)  High Grey Level Zone Emphasis (HGLZE)  Small Area Low Grey Level Emphasis (SALGLE)  Small Area High Grey Level Emphasis (SAHGLE)  Large Area Low Grey Level Emphasis (LALGLE)  Large Area High Grey Level Emphasis (LAHGLE) |
| Third | Grey level run length matrix (GLRLM) | Short Run Emphasis (SRE)  Long Run Emphasis (LRE)  Grey Level Non-Uniformity (GLN)  Grey Level Non-Uniformity Normalized (GLNN)  Run Length Non-Uniformity (RLN)  Run Length Non-Uniformity Normalized (RLNN)  Run Percentage (RP)  Grey Level Variance (GLV)  Run Variance (RV)  Run Entropy (RE)  Low Grey Level Run Emphasis (LGLRE)  High Grey Level Run Emphasis (HGLRE)  Short Run Low Grey Level Emphasis (SRLGLE)  Short Run High Grey Level Emphasis (SRHGLE)  Long Run Low Grey Level Emphasis (LRLGLE)  Long Run High Grey Level Emphasis (LRHGLE) |
| Third | Neighbouring grey tone difference matrix (NGTDM) | Coarseness  Contrast  Busyness  Complexity  Strength |
| Third | Grey level dependence matrix (GLDM) | Small Dependence Emphasis (SDE)  Large Dependence Emphasis (LDE)  Grey Level Non-Uniformity (GLN)  Dependence Non-Uniformity (DN)  Dependence Non-Uniformity Normalized (DNN)  Grey Level Variance (GLV)  Dependence Variance (DV)  Dependence Entropy (DV)  Low Grey Level Emphasis (LGLE)  High Grey Level Emphasis (HGLE)  Small Dependence Low Grey Level Emphasis (SDLGLE)  Small Dependence High Grey Level Emphasis (SDHGLE)  Large Dependence Low Grey Level Emphasis (LDLGLE)  Large Dependence High Grey Level Emphasis (LDHGLE) |

**S8**. Data analysis.

To estimate the robustness of features to MR change, the Concordance Correlation Coefficient (CCC) and the Intra-class Correlation Coefficient (ICC) were used ^1^.

ICC estimates the magnitude of the relationship between variables. ICC also considers rater bias. It ranges between 0 and 1, indicating null and perfect reproducibility respectively. In order to determine ICC for machine variability, which reflects feature variations for the same subject, a two-way mixed effect model, defined by McGraw and Wong ^2^, was used:

$$ICC=\frac{{MS}_{R}-{MS}_{E}}{{MS}_{R}+\left( k-1 \right){MS}_{E}}$$

Where ${MS}_{R}$ corresponds to the mean square for rows (subjects), ${MS}_{E}$ to the mean square for error and $k$ to the number of measurements.

CCC is another measure of agreement which, unlike ICC, does not assume a common mean for machine difference at the outset. Lin ^3^ defines it as follows:

$$CCC=\frac{2\rho\sigma_{x}\sigma_{y}}{\sigma_{x}^{2}+\sigma_{y}^{2}+\left( \mu_{x}-\mu_{y} \right)^{2}}$$

Where $\mu_{x}$ and $\mu_{y}$ are the means for the two variables (here the radiomics features), $\sigma_{x}$ and $\sigma_{y}$ are the corresponding variances and $\rho$ is the correlation coefficient between the two variables.
